# Access of transgender people and transvestites to pre-exposure prophylaxis for HIV in Brazil: a descriptive study, 2018-2023

**DOI:** 10.1590/S2237-96222024v33e2024322.especial.en

**Published:** 2025-01-10

**Authors:** Aline Pilon Mauricio da Silva, Isabela Ornelas Pereira, Tatianna Meireles D. Alencar, Beatriz Brittes Kamiensky, Marihá Camelo Madeira de Moura, Francisco Álisson Paula de França, Thiago Cherem Morelli, Ana Roberta Pati Pascom

**Affiliations:** 1 Ministério da Saúde, Secretaria de Vigilância em Saúde e Ambiente, Brasília, DF, Brasil

**Keywords:** Profilaxis Pre-Exposición, Personas Transgénero, VIH, Estudios Transversales, Pre-Exposure Prophylaxis, Transgender People, HIV, Cross-Sectional Studies

## Abstract

**Objectives:**

To analyze access to pre-exposure prophylaxis (PrEP) for HIV in Brazil, comparing transgender and cisgender populations.

**Methods:**

This was a descriptive study using data from the Medication Logistics Control System (*Sistema de Controle Logístico de Medicamentos* - SICLOM), related to the monitoring of PrEP between January 2018 and December 2023.

**Results:**

During the period analyzed, 149,022 people initiated PrEP, of whom 139,423 (94%) were cisgender and 9,599 (6%) were transgender. 54% cisgender and 46% transgender people were of White/Asian skin color, 67% cisgender and 48% transgender people had 12 or more years of study, and 40% cisgender and 52% transgender people were aged 15 to 29 years.

**Conclusion:**

The data showed disparities in access to PrEP, with the transgender population still underrepresented. Expanding targeted strategies may mitigate specific barriers faced by this population and promote greater equity in access to prophylaxis.

## Study contributions

**Main results:**

Access to pre-exposure prophylaxis (PrEP) for HIV was limited among transgender people and transvestite. At national level, access revealed vulnerabilities and inequalities.

**Implications for services:**

There is a need to raise awareness among services to reduce access barriers and expand access to PrEP for this population.

**Perspectives:**

It is necessary to expand equitable access to PrEP, addressing stigma and discrimination.

## RESUMEN

**Objetivos:**

Analizar el acceso a la profilaxis previa a la exposición al VIH (PrEP) en Brasil, comparando el acceso entre las poblaciones trans y cis.

**Métodos:**

Estudio descriptivo con datos provenientes del Sistema de Control Logístico de Medicamentos (Siclom), relativos al monitoreo de la PrEP entre enero de 2018 y diciembre de 2023.

**Resultados:**

En el período analizado, 149.022 personas iniciaron PrEP, de las cuales 139.423 (94%) eran cis y 9.599 (6%) trans. Se registró que el 54% de la población cis y el 46% de la población trans eran blancas/amarillas. Además, el 67% de las personas cis y el 48% de las trans tenían 12 o más años de escolaridad, mientras que el 40% de las personas cis y el 52% de las trans tenían entre 15 y 29 años.

**Conclusión:**

Los datos revelaron disparidades en el acceso a la PrEP, con la población trans aún subrepresentada. La ampliación de estrategias dirigidas podría mitigar las barreras específicas que enfrenta esta población y promover una mayor equidad en el acceso a la profilaxis.

## INTRODUCTION

In Brazil, HIV infection is high in certain population subgroups. Among these, the most prominent are gay men and other men who have sex with men, sex workers, people who inject drugs and specially transvestites and transgender people, who are among the most vulnerable to HIV infection. In some Brazilian capitals, a study estimated the prevalence of HIV in transvestites and transgender women to range between 16.9% and 36.7%, while in the general population this rate was 0.4%.^1^

According to Stutterheim et al.,^2^ transgender women and transvestites are up to 66 times more likely to acquire HIV compared to other individuals aged 15 or older. In addition, transgender men, a group still underrepresented in the literature, are seven times more likely to contract HIV than other individuals^2^. These data underscore the urgency of interventions aimed at this population, such as expanding access to pre -exposure prophylaxis (PrEP) for HIV prevention.

PrEP is a safe and effective biomedical strategy for preventing HIV.^3^ Studies have shown that locations with high coverage and use of prophylaxis have demonstrated a reduction in new cases and incidence of HIV, highlighting the effectiveness of implementing and expanding this strategy.^4-6^ In Brazil, oral PrEP, containing co-formulated antiretroviral drugs – tenofovir desoproxil fumarate (TDF) and emtricitabine (FTC) – was incorporated into the Brazilian National Health System (*Sistema Único de Saúde* - SUS) in 2017.^7^ Since then, over 900 drug dispensing units have offered PrEP across the country, reaching approximately 149,000 users.^8^

Although PrEP is available through the SUS, transgender people and transvestites remain underrepresented among PrEP users. Only 0.3% of users are transvestites and 2.9% are transgender women, which may highlight significant barriers to access. These difficulties may be associated with social and structural factors, such as LGBTphobia, transphobia, racism and stigmatization, which exacerbate the exclusion of these populations from healthcare services. This underrepresentation highlights the need for specific and inclusive strategies to mitigate these barriers and ensure greater access to PrEP for these vulnerable populations.^9-13^

To reduce HIV incidence and achieve its elimination as a public health problem by 2030,^14^ it is essential to understand the specific needs of the most vulnerable populations, especially among transgender people and transvestites. Given that, there is an urgent need to assess the current scenario of PrEP users and guide public health policies that promote equity. Thus, the objective of this study was to analyze access to PrEP by comparing transgender and cisgender populations.

## METHODS

A descriptive cross-sectional study was conducted to analyze Siclom⁸ data related to PrEP monitoring in Brazil, from January 2018 to December 2023. All registered users who received at least one PrEP dispensing during this period were included in the analysis. The information was extracted from the national registration forms filled out by users when obtaining PrEP at public pharmacies under the SUS. These forms include data on sexual orientation and gender identity, which characterize individuals as: gay men, men who have sex with men, transvestites, transgender women, transgender men, cisgender women, cisgender and transgender heterosexual men, and non-binary people. For the purposes of this study, these groups were condensed into two: (1) transgender people, which included transvestites, transgender women, transgender men, and non-binary people; and (2) cisgender people, which included gay men and men who have sex with cisgender men, cisgender women, and cisgender heterosexual men.

The sociodemographic data analyzed included age group, education level, and race/skin color. The age group was calculated based on users’ age at the time of PrEP dispensing, and distributed into six groups: 15 to 17 years, 18 to 24 years, 25 to 29 years, 30 to 39 years, 40 to 49 years, and 50 years or older. Education level was classified into four categories: ≥3 years, 4 to 7 years, 8 to 11 years, and 12 or more years of study. The self-declared race/skin color variable was divided into four categories: mixed-race, Black, Indigenous, and White/Asian, with the last two being grouped together due to the small number of Asian individuals and the sociodemographic similarities between these groups in the Brazilian context.

Absolute and relative frequencies were calculated for each gender identity group and for sociodemographic variables, separately for transgender and cisgender people. The users’ access to health services was classified according to the type of healthcare system, with public services defined as those provided by SUS units and private services offered by the supplementary healthcare system. Analyses considered the absolute and relative frequencies of public and private healthcare visits for each gender identity group, with proportions calculated in relation to the total number of recorded visits.

Statistical analyses were performed using the Python programming language (version 3.11.4) and the Pandas library, due to the capacity of these tools to process large datasets and facilitate data manipulation and interpretation. To calculate the p-value, the Person chi -square test was used. Results with an error of less than 0.05 were considered statistically significant. All data used are secondary and were anonymized to ensure user confidentiality. Prior to the analysis, the Ministry of Health (MS) assigned a unique code to each case, in order to protect confidentiality and prevent individual identification. The data are also publicly available through the PrEP dashboard⁸ and in the database made available by Siclom.^16^ The study was conducted in accordance with Resolution No. 466 of the National Health Council, which ensured compliance with ethical principles for research involving human subjects.

## RESULTS

Between January 2018 and December 2023, a total of 149,022 people initiated PrEP, of whom 139,423 (93.6%) were cisgender people and 9,599 (6.4%) were transgender people. Over the years, there was an observed increase in the number of people starting PrEP, accompanied by a slight decrease in the percentage of cisgender individuals and an increase in the proportion of transgender individuals. Cisgender people increased from 7,773 in 2018 to 51,425 in 2023, a 562% rise. Transgender people ranged from 427 in 2018 to 4,153 in 2023, representing an 873% increase. In 2023, the highest number of users who initiated PrEP was recorded, with over 51,000 cisgender people and more than 4,000 transgender people, which represented 92.5% and 7.5% of the total number of people who initiated prophylaxis that year, respectively (Table 1).

**Table 1:** Distribution of cisgender and transgender people who initiated pre-exposure prophylaxis for HIV in Brazil between 2018 and 2023

Year	Cisgender (%)	Transgender (%)
2018	7,773 (94.8)	427 (5.2)
2019	11,848 (94.5)	689 (5.5)
2020	12,410 (95.4)	593 (4.6)
2021	21,961 (94.8)	1,201 (5.2)
2022	34,006 (93.1)	2,536 (6.9)
2023	51,425 (92.5)	4,153 (7.5)
Total	139,423 (93.6)	9,599 (6.4)

The largest proportion of individuals who initiated PrEP between 2018 and 2023 was in the 30-39 age group, both for cisgender (38.2%) and transgender people (32.0%), followed by 25-29 age group, in both populations (Table 2). Half of the transgender people using PrEP were aged 15 to 29 years (52.0%), while among cisgender users, this age group accounted for 40.2%. The proportion of individuals aged 50 years and older was lower, at 6.0% for cisgender people and 4.0% for transgender people (p-value<0.001).

**Table 2:** Sociodemographic characteristics of cisgender and transgender people who initiated pre-exposure prophylaxis for HIV in Brazil between 2018 and 2023

Variables	Cisgender (%)	Transgender (%)	p-value^a^
**Age group (years)**			<0.001
<18	319 (0.2)	66 (0.7)	
18-24	21,435 (15.4)	2,303 (24.0)	
25-29	35,056 (25.1)	2,624 (27.3)	
30-39	53,305 (38.2)	3,074 (32.0)	
40-49	20,969 (15.0)	1,146 (11.9)	
≥50s	8,338 (6.0)	386 (4.0)	
Total	139,422 (100.0)	9,599 (100.0)	
**Education level (years)**			<0.001
0-3	2,069 (1.5)	229 (2.4)	
4-7	6,203 (4.5)	990 (10.3)	
8-11	37,289 (26.8)	3,808 (39.7)	
≥12	93,823 (67.3)	4,569 (47.6)	
Total	139,384 (100.0)	9,596 (100.0)	
**Race/skin color**			<0.001
White and Asian	75,721 (54.3)	4,425 (46.1)	
Mixed-race	45,329 (32.5)	3,440 (35.8)	
Black	17,796 (12.8)	1,645 (17.1)	
Indigenous	558 (0.4)	89 (0.9)	
Total	139,404 (100.0)	9,599 (100.0)	

a) Pearson’s chi-square test.

Most people who initiated PrEP had 12 or more years of study, with a higher proportion among transgender people (47.6%). The proportion of people with 8-11 years of study was also significant, particularly among transgender people (39.7%), who had lower education levels (p-value<0.001), suggesting that education levels among people who initiated PrEP differed significantly between cisgender and transgender people (Table 2).

The largest proportion of cisgender people initiating PrEP were of White or Asian (54.3%), mixed-race (32.5%) and Black (12.8%) race/skin color. Among transgender users, the majority were Black and mixed-race (52.9%) (p-value<0.001).

The majority of PrEP services were provided through the public health system, both for cisgender (90.5%) and transgender people (92.8%). A higher proportion of cisgender people accessed PrEP through private services (9.5%) compared to transgender people (7.2%).

Private sector services showed a higher concentration of individuals aged 30 to 39 (51%), with 12 or more years of study (86.5%), and who self-declared as Asian or White (71.3%). The largest group in the public sector also consisted of individuals aged 30 and 39 years (38%), followed by those aged 25 to 29 years (25.8%). The educational distribution was similar to that of the public sector (Table 4).

**Table 3:** Care provided by pre-exposure prophylaxis for HIV according to type of service and gender, Brazil, 2018-2023

Service	Cisgender (%)	Transgender (%)
Private	41,623 (9.5)	1,618 (7.2%)
Public	396,419 (90.5)	20,816 (92.8)
Total	438,042 (100.0)	22,434 (100.0)

**Table 4:** Sociodemographic characteristics of people who initiated pre-exposure prophylaxis for HIV according to type of service and gender, Brazil, 2018-2023

Variables	Private (%)	Public (%)	p-value^a^
**Age group (years)**			<0.001
<18	-	71 (0.3)	
18-24	117 (7.2)	3,264 (15.7)	
25-29	351 (21.7)	5,374 (25.8)	
30-39	825 (51.0)	7,918 (38.0)	
40-49	277 (17.1)	3,012 (14.5)	
≥50s	48 (3.0)	1,177 (5.7)	
Total	1.618 (100.0)	20,816 (100.0)	
**Education level (years)**			<0.001
≤3	28 (1.7)	475 (2.3)	
4-7	21 (1.3)	1,748 (8.4)	
8-11	170 (10.5)	8,311 (39.9)	
≥12	1,399 (86.5)	10,280 (49.4)	
Total	1.618 (100.0)	20,814 (100.0)	
**Race/skin color**			<0.001
White and Asian	1,153 (71.3)	9,807 (47.1)	
Mixed-race	3 (0.2)	210 (1.0)	
Black	316 (19.5)	7,648 (36.7)	
Indigenous	146 (9.0)	3,151 (15.1)	
Total	1.618 (100.0)	20.816 (100.0)	

## DISCUSSION

Providing pre-exposure prophylaxis (PrEP) in Brazil is a crucial strategy in the response to HIV, especially for more vulnerable populations, such as transvestites and transgender people. The study data showed that, despite the increase in their inclusion between 2018 and 2023, these populations remain underrepresented, accounting for only 6% of users. These data reflect significant and persistent barriers, such as transphobia and stigma, which hinder continuous access to healthcare services. Furthermore, according to Poteat et al.,^15^ these populations face additional barriers, including discrimination and social exclusion, which not only increase their risk of infection but also limit access to preventive services. The integration of inclusive and welcoming policies is essential to mitigate these risks and ensure health equity.

Prejudice in healthcare services and the lack of training for professionals are highlighted as critical factors that discourage transgender people from seeking preventive care.^10^ The inclusion of health education strategies that consider gender diversity is essential to reduce these gaps. In the context of this study, while the provision of PrEP through the SUS has expanded, it is still insufficient to overcome cultural and structural barriers faced by this population.

Almost half of transgender people using PrEP identify as White or Asian, one third are mixed-race, and less than 20% are Black. Barriers to accessing PrEP are more pronounced among Black people and those with lower level of education, highlighting the importance of public policies that ensure racial and social equity.^9^

Slightly more than half of transgender individuals initiating PrEP were aged 15 to 29 in this study, consistent with the increasing incidence of HIV infections among young people in this age group in Brazil.^16^ Retaining transgender users in PrEP programs is a critical challenge, suggesting the need for innovative strategies to ensure treatment continuity.^17^

Approximately half of transgender people using PrEP had 12 or more years of study, but a significant proportion had lower level of education. The inclusion of peer-led educational strategies is considered essential to improve adherence and continuity of PrEP use, especially among populations facing multiple forms of social exclusion.^18^

Another relevant point is the provision of PrEP in both the public and private sectors. Although most care is provided by the SUS, expanding access through private services, as planned by the Ministry of Health,^19-20^ can contribute to greater reach and accessibility. Integration of services and decentralization of provision are essential to serve the most vulnerable populations. Collaboration between public and private systems is essential to ensure continuity of care and adherence to PrEP among key populations, such as transgender women.^13^ Prescription by non-physician healthcare professionals, such as nurses and pharmacists, is also strategic to expand coverage and reduce barriers.^12^ A positive impact of this approach has been observed in implementation projects in Latin America.^12^

Overcoming the barriers faced by transgender people requires an integrated approach that involves not only offering PrEP but also promoting inclusive and discrimination-free healthcare environments. Effective public prevention policies must prioritize equity and address stigma to ensure that all people at risk of infection can access and benefit from PrEP.^14^

An important limitation of this study is that it relies on secondary and anonymized data from SICLOM, presented through the PrEP dashboard,^8^ which prevents a more in-depth qualitative analysis of the reasons that lead to low adherence and access to PrEP among transgender people and transvestite. Although the study identifies relevant disparities, it does not provide detailed information on the social, cultural and structural barriers faced by this population. In addition, the lack of census data on the trans population in Brazil makes it difficult to accurately estimate PrEP coverage, which is essential to assess the effectiveness of public policies and the reach of this preventive method.^21^

Future research could benefit from qualitative and mixed-methods approaches to gain a deeper understanding of the factors that affect trans people’s access to and retention in PrEP programs. Research involving interviews with health professionals and users could reveal aspects related to discrimination, inadequate care and lack of information that are not captured by quantitative data.^19^ In addition, studies that analyze the impact of specific interventions, such as PrEP prescription by pharmacists and nurses, could contribute to expanding implementation strategies. Another promising line of research would be to explore the introduction of new forms of long-acting PrEP, aiming to increase adherence among vulnerable populations.^22^

Although this study has provided valuable contributions by revealing the underrepresentation of transgender individuals among PrEP users in Brazil, it also reinforces the need for more inclusive and targeted policies. Expanding access to PrEP is essential to promote equity and reduce HIV incidence in this population, especially considering the high prevalence rates of the virus among trans people. Therefore, expanding prevention strategies and addressing structural and social barriers are critical steps to ensure that PrEP more effectively benefits those who need it most.
