# Supplementary material for: Access of transgender people and transvestites to pre-exposure prophylaxis for HIV in Brazil: a descriptive study, 2018-2023
Source: Epidemiol Serv Saude. 2025 Jan 10;33(spe1):e2024322. doi: 10.1590/S2237-96222024v33e2024322.especial.en (PMC11734650; doi:10.1590/S2237-96222024v33e2024322.especial.en)
Supplement: Translation

# Translation: Acesso de pessoas trans e travestis à profilaxia pré-exposição ao HIV no Brasil: estudo descritivo, 2018-2023

**DOI:** 10.1590/S2237-96222024v33e2024322.especial.pt

**Authors:** Aline Pilon Mauricio da Silva, Isabela Ornelas Pereira, Tatianna Meireles D. Alencar, Beatriz Brittes Kamiensky, Marihá Camelo Madeira de Moura, Francisco Álisson Paula de França, Thiago Cherem Morelli, Ana Roberta Pati Pascom

**Affiliations:** 1 Ministério da Saúde, Secretaria de Vigilância em Saúde e Ambiente, Brasília, DF, Brasil

**Keywords:** Profilaxia Pré-Exposição, Pessoas Transgênero, HIV, Estudos Transversais

## Abstract

**Objetivos:**

Analisar o acesso à profilaxia pré-exposição ao HIV (PrEP) no Brasil, comparando-o entre população trans e cis.

**Métodos:**

Estudo descritivo com dados oriundos do Sistema de Controle Logístico de Medicamentos (Siclom), referentes ao monitoramento da PrEP entre janeiro de 2018 e dezembro de 2023.

**Resultados:**

No período analisado, 149.022 pessoas iniciaram PrEP, sendo 139.423 (94%) cis e 9.599 (6%) trans. Foram registradas 54% cis e 46% trans brancas/amarelas, 67% cis e 48% trans com 12 anos ou mais de escolaridade e 40% cis e 52% trans na faixa etária entre 15 e 29 anos.

**Conclusão:**

Os dados revelaram disparidades no acesso à PrEP, com a população trans ainda subrepresentada. A ampliação de estratégias direcionadas pode mitigar barreiras específicas enfrentadas por essa população e promover maior equidade no acesso à profilaxia.

## Contribuições do estudo

**Principais resultados:**

O acesso à profilaxia pré-exposição ao HIV (PrEP) mostrou-se limitado em pessoas trans e travestis. Nacionalmente, o acesso evidenciou vulnerabilidades e desigualdades.

**Implicações para os serviços:**

É necessário sensibilizar os serviços para reduzir barreiras de acesso e ampliar o acesso a PrEP para alcance dessa população.

**Perspectivas:**

É necessário expandir a oferta de PrEP com equidade e enfrentamento ao estigma e discriminação.

## INTRODUÇÃO

No Brasil, a infecção por HIV mostra-se elevada em determinados subgrupos populacionais. Entre esses, destacam-se *gays* e outros homens que fazem sexo com homens, profissionais do sexo, usuários de drogas injetáveis e, em especial, travestis e pessoas transexuais, que compõem o grupo mais vulnerável à infecção pelo HIV. Em algumas capitais brasileiras, um estudo estimou a prevalência de HIV em travestis e mulheres trans entre 16,9% e 36,7%, enquanto na população geral essa taxa foi de 0,4%.^1^

De acordo com Stutterheim et al.,^2^ mulheres trans e travestis possuem uma probabilidade de até 66 vezes maior de contrair HIV em comparação a outros indivíduos com 15 anos ou mais. Além disso, homens trans, um grupo ainda pouco estudado na literatura, apresentam uma chance sete vezes superior de adquirir HIV em relação a outros indivíduos.^2^ Esses dados reforçam a urgência de intervenções voltadas para essa população, como a ampliação da oferta da profilaxia pré-exposição (PrEP) de risco ao HIV.

A PrEP é uma estratégia biomédica segura e eficaz na prevenção ao HIV.^3^ Estudos indicaram que locais com alta cobertura e uso da profilaxia têm mostrado redução nos novos casos e na incidência do HIV, o que evidencia a efetividade da implementação e da expansão dessa estratégia.^4^^-^^6^ No Brasil, a PrEP oral, composta por medicamentos antirretrovirais coformulados – fumarato de tenofovir desoproxila (TDF) e entricitabina (FTC) – foi incorporada ao Sistema Único de Saúde (SUS) em 2017.^7^ Desde então, mais de 900 unidades dispensadoras de medicamentos oferecem PrEP em todo o país, alcançando cerca de 149.000 usuários.^8^

Embora a PrEP esteja disponível no SUS, pessoas trans e travestis permanecem subrepresentadas entre os usuários dessa profilaxia. Apenas 0,3% dos usuários são travestis e 2,9% são mulheres trans, o que pode evidenciar importantes barreiras de acesso. Essas dificuldades podem estar relacionadas com fatores sociais e estruturais, como LGBTfobia, transfobia, racismo e estigmatização, que agravam a exclusão dessas populações dos serviços de saúde. Essa subrepresentação ressalta a necessidade de estratégias específicas e inclusivas para mitigar essas barreiras e garantir maior acesso à PrEP para essas populações vulneráveis.^9-13^

Para reduzir a incidência de HIV e alcançar sua eliminação como um problema de saúde pública até 2030,^14^ é essencial compreender as especificidades das populações mais vulneráveis, especialmente entre as pessoas trans e travestis. Com essa compreensão, urge a necessidade de se verificar o panorama atual dos usuários de PrEP e direcionar políticas públicas de saúde que promovam a equidade. Assim, o objetivo deste estudo é analisar o acesso à PrEP, comparando as populações trans e cis.

## MÉTODOS

Foi realizado um estudo transversal, com abordagem descritiva, para analisar dados Siclom,^8^ referentes ao monitoramento da PrEP no Brasil, no período de janeiro de 2018 a dezembro de 2023. Foram incluídos na análise todos os usuários cadastrados que receberam ao menos uma dispensação de PrEP durante esse período. As informações utilizadas foram extraídas dos formulários de cadastro nacional preenchidos pelos usuários ao retirarem a PrEP em farmácias do SUS. Esses formulários incluem dados sobre orientação sexual e identidade de gênero, que caracteriza os indivíduos como: *gays*, homens que fazem sexo com homens, travestis, mulheres trans, homens trans, mulheres cis, homens heterossexuais cis e trans, e pessoas não binárias. Para fins deste estudo, esses grupos foram condensados em dois: (1) pessoas trans, que incluiu travestis, mulheres trans, homens trans e não binárias; e (2) pessoas cis, que incluiu *gays* e homens que fazem sexo com homens cis, mulheres cis e homens heterossexuais cis.

Os dados sociodemográficos analisados incluíram faixa etária, escolaridade e raça/cor. A faixa etária foi calculada com base na idade dos usuários no momento da dispensação da PrEP, sendo distribuída em seis grupos: 15 a 17 anos, 18 a 24 anos, 25 a 29 anos, 30 a 39 anos, 40 a 49 anos e 50 anos ou mais. A escolaridade foi classificada em quatro categorias: ≥3 anos, de 4 a 7 anos, de 8 a 11 anos e 12 ou mais anos de estudo. A variável raça/cor autodeclarada foi dividida em quatro categorias: parda, preta, indígena e branca/amarela, sendo as duas últimas agrupadas devido ao pequeno número de indivíduos de cor amarela e à similaridade sociodemográfica entre esses grupos no contexto brasileiro.

As frequências absolutas e relativas foram calculadas para cada grupo de identidade de gênero e para as variáveis sociodemográficas, separadamente para pessoas trans e cis. O atendimento dos usuários foi classificado conforme a origem do serviço de saúde, sendo considerado público quando realizado em unidades do SUS e privado quando oferecido por serviços do sistema suplementar. As análises consideraram as frequências absolutas e relativas dos atendimentos públicos e privados em cada grupo de identidade de gênero, com as proporções calculadas em relação ao total de atendimentos registrados.

As análises estatísticas foram realizadas utilizando a linguagem de programação Python (versão 3.11.4) e a biblioteca Pandas, devido à capacidade dessas ferramentas em processar grandes volumes de dados e facilitar a manipulação e interpretação dos resultados. Para calcular o p-valor, utilizou-se o teste de qui-quadro de Person. Considerou-se resultado estatisticamente significativos aqueles cujo erro foi inferior a 0,05. Todos os dados utilizados são secundários e foram anonimizados para garantir a confidencialidade dos usuários. Antes da análise, o Ministério da Saúde (MS) atribuiu um código único a cada caso, de forma a resguardar o sigilo e a evitar a identificação dos indivíduos. Os dados também estão disponíveis publicamente no painel PrEP⁸ e no banco de dados disponibilizado pelo Siclom.^16^ O estudo foi conduzido em conformidade com a Resolução nº 466 do Conselho Nacional de Saúde, o que garantiu a observância dos princípios éticos na pesquisa com seres humanos.

## RESULTADOS

Entre janeiro de 2018 e dezembro de 2023, 149.022 pessoas iniciaram PrEP, ou seja, sendo 139.423 (93,6%) pessoas cis e 9.599 (6,4%) pessoas trans. Pode-se observa um aumento, ao longo dos anos, no número de pessoas que iniciaram a PrEP com uma ligeira diminuição percentual na proporção de pessoas cis e um aumento na proporção de pessoas trans. As pessoas cis passaram de 7.773 em 2018 para 51.425 em 2023, um aumento de 562%. As pessoas trans passaram de 427 em 2018 para 4.153 em 2023, um aumento de 873%. Em 2023, foi registrado o maior número de usuários que iniciaram PrEP, com mais de 51 mil pessoas cis e mais de 4 mil pessoas trans, o que representou 92,5% e 7,5% do total de pessoas que iniciaram a profilaxia naquele ano, respectivamente (Tabela 1).

**Tabela 1:** Distribuição de pessoas cisgênero e transgênero que iniciaram a profilaxia préexposição ao HIV no Brasil entre 2018 a 2023

Ano	Cisgênero (%)	Transgênero (%)
2018	7.773 (94,8)	427 (5,2)
2019	11.848 (94,5)	689 (5,5)
2020	12.410 (95,4)	593 (4,6)
2021	21.961 (94,8)	1.201 (5,2)
2022	34.006 (93,1)	2.536 (6,9)
2023	51.425 (92,5)	4.153 (7,5)
Total	139.423 (93,6)	9.599 (6,4)

Maior proporção de pessoas que iniciou PrEP entre 2018 e 2023 está na faixa etária de 30 a 39 anos, tanto para pessoas cis (38,2%) quanto para pessoas trans (32,0%), entre 25 e 29 anos, nas duas populações (Tabela 2). Metade das pessoas trans que fazem uso de PrEP tem de 15 a 29 anos (52,0%), enquanto nesta mesma faixa etária, usuários cis representam 40,2%. A proporção de indivíduos com 50 anos ou mais foi menor, 6,0% em pessoas cis e 4,0% em pessoas trans (p-valor<0,001).

**Tabela 2:** Características sociodemográficas de pessoas cisgênero e transgênero que iniciaram a profilaxia pré-exposição de risco ao HIV no Brasil entre 2018 a 2023

Variáveis	Cisgênero (%)	Transgênero (%)	p-valor^a^
**Faixa etária (anos)**			<0,001
<18	319 (0,2)	66 (0,7)	
18-24	21.435 (15,4)	2.303 (24,0)	
25-29	35.056 (25,1)	2.624 (27,3)	
30-39	53.305 (38,2)	3.074 (32,0)	
40-49	20.969 (15,0)	1.146 (11,9)	
≥50s	8.338 (6,0)	386 (4,0)	
Total	139.422 (100,0)	9.599 (100,0)	
**Escolaridade (anos)**			<0,001
0-3	2.069 (1,5)	229 (2,4)	
4-7	6.203 (4,5)	990 (10,3)	
8-11	37.289 (26,8)	3.808 (39,7)	
≥12	93.823 (67,3)	4.569 (47,6)	
Total	139.384 (100,0)	9.596 (100,0)	
**Raça/cor da pele**			<0,001
Branca e amarela	75.721 (54,3)	4.425 (46,1)	
Parda	45.329 (32,5)	3.440 (35,8)	
Preta	17.796 (12,8)	1.645 (17,1)	
Indígena	558 (0,4)	89 (0,9)	
Total	139.404 (100,0)	9.599 (100,0)	

a) Teste qui-quadrado de Pearson.

A maioria das pessoas que iniciou a PrEP tinha 12 ou mais anos de estudo, maior entre pessoas trans (47,6%). A proporção de pessoas com 8-11 anos de educação também foi considerável, particularmente entre pessoas trans (39,7%), que foi tiveram menor escolaridade (p-valor<0,001), o que sugere que escolaridade das pessoas que iniciaram PrEP diferem significativamente entre pessoas cis e trans (Tabela 2).

A maior proporção das pessoas cis que iniciou a PrEP tinha raça/cor da pele branca ou amarela (54,3%), parda (32,5%) e preta (12,8%). Entre usuárias trans, a maioria era pretas e pardas (52,9%) (p-valor<0,001). 

A maior parte dos atendimentos de PrEP foi realizada no serviço público, tanto para pessoas cis (90,5%), quanto para pessoas trans (92,8%). Mais pessoas cis tiveram atendimento da PrEP no serviço privado (9,5%) em relação às pessoas trans (7,2%). 

**Tabela 3:** Atendimentos de profilaxia pré-exposição de risco ao HIV de acordo com o tipo de serviço e gênero, Brasil, 2018-2023

Serviço	Cisgênero (%)	Transgênero (%)
Privado	41.623 (9,5)	1.618 (7,2%)
Público	396.419 (90,5)	20.816 (92,8)
Total	438.042 (100,0)	22.434 (100,0)

Os atendimentos no setor privado tiveram maior concentração de pessoas de 30 a 39 anos (51%), com 12 ou mais anos de estudo (86,5%) e autodeclaradas amarela ou branca (71,3%). O maior grupo atendido no setor público também tinha entre 30-39 anos (38%), seguido por 25-29 anos (25,8%). A distribuição da escolaridade mostrou-se semelhante a do serviço público (Tabela 4).

**Tabela 4:** Características sociodemográficas de pessoas que iniciaram a profilaxia pré-exposição de risco ao HIV de acordo com o tipo de serviço e gênero, Brasil, 2018-2023

Variáveis	Privado (%)	Público (%)	p-valor^a^
**Faixa etária (anos)**			<0,001
<18	-	71 (0,3)	
18-24	117 (7,2)	3.264 (15,7)	
25-29	351 (21,7)	5.374 (25,8)	
30-39	825 (51,0)	7.918 (38,0)	
40-49	277 (17,1)	3.012 (14,5)	
≥50s	48 (3,0)	1.177 (5,7)	
Total	1.618 (100,0)	20.816 (100,0)	
**Escolaridade (anos)**			<0,001
≤3	28 (1,7)	475 (2,3)	
4-7	21 (1,3)	1.748 (8,4)	
8-11	170 (10,5)	8.311 (39,9)	
≥12	1.399 (86,5)	10.280 (49,4)	
Total	1.618 (100,0)	20.814 (100,0)	
**Raça/cor da pele**			<0,001
Branca e amarela	1.153 (71,3)	9.807 (47,1)	
Parda	3 (0,2)	210 (1,0)	
Preta	316 (19,5)	7.648 (36,7)	
Indígena	146 (9,0)	3.151 (15,1)	
Total	1.618 (100,0)	20.816 (100,0)	

## DISCUSSÃO

A oferta de profilaxia PrEP no Brasil é uma estratégia essencial na resposta ao HIV, especialmente para populações em maior vulnerabilidade, como travestis e pessoas trans. Os dados do estudo demonstraram que, apesar do crescimento na sua inclusão entre 2018 e 2023, essas populações continuam subrepresentadas, correspondendo a apenas 6% dos usuários. Esse dado reflete barreiras significativas e persistentes, como transfobia e estigma, que dificultam o acesso contínuo aos serviços de saúde. Além disso, segundo Poteat et al.,^15^ essas populações enfrentam barreiras adicionais, incluindo discriminação e exclusão social, que não apenas aumentam seu risco de infecção, mas também dificultam o acesso a serviços preventivos. A integração de políticas inclusivas e acolhedoras é essencial para mitigar esses riscos e assegurar a equidade na saúde.

O preconceito nos serviços de saúde e a falta de capacitação de profissionais são apontados como fatores críticos que afastam pessoas trans do atendimento preventivo.^10^ A inclusão de estratégias de educação em saúde que considerem a diversidade de gênero é essencial para reduzir essas lacunas. No contexto deste estudo, a oferta da PrEP no SUS, embora tenha se expandido, ainda não é suficiente para superar barreiras culturais e estruturais enfrentadas por essa população.

Quase metade das pessoas trans usuárias de PrEP se identificam como brancas ou amarelas, um terço foi composto por pessoas pardas, e menos de 20% por pessoas pretas. Barreiras ao acesso à PrEP são mais pronunciadas entre indivíduos negros e de baixa escolaridade, evidenciando a importância de políticas públicas que assegurem equidade racial e social.^9^

Pouco mais da metade das pessoas trans que iniciaram a PrEP tinham entre 15 e 29 anos nesta pesquisa, consistente com aumento da incidência de infecções por HIV entre jovens nessa faixa etária no Brasil.^16^ A retenção de usuários trans em programas de PrEP é um desafio crítico, sugerindo a necessidade de estratégias inovadoras para garantir a continuidade do tratamento.^17^

Cerca de metade das pessoas trans usuárias de PrEP tinham estudado 12 anos ou mais, mas proporção significativa apresentou escolaridade mais baixa. A inclusão de estratégias educativas conduzidas por pares é considerada fundamental para melhorar a adesão e a continuidade do uso da PrEP, especialmente em populações que enfrentam múltiplas formas de exclusão social.^18^

Outro ponto relevante é a oferta de PrEP no setor público e privado. Embora a maior parte dos atendimentos ocorra no SUS, a ampliação da oferta por meio de serviços privados, como previsto pelo Ministério da Saúde,^19-20^ pode contribuir para maior alcance e acessibilidade. A integração de serviços e a descentralização da oferta são essenciais para atender às populações em maior vulnerabilidade. A colaboração entre sistemas públicos e privados deve ocorrer para garantir a continuidade do cuidado e a adesão à PrEP em populações chave, como mulheres trans.^13^ A prescrição por profissionais de saúde não médicos, como enfermeiros e farmacêuticos é também estratégica ampliar a cobertura e reduzir barreiras.^12^ Impacto positivo dessa abordagem foi observado em projetos de implementação na América Latina.^12^

A superação das barreiras enfrentadas por pessoas trans requer uma abordagem integrada que envolva não apenas a oferta de PrEP, mas também a promoção de ambientes de saúde inclusivos e livres de discriminação. Políticas públicas de prevenção eficazes devem priorizar a equidade e o enfrentamento ao estigma para garantir que todas as pessoas em risco de infecção possam acessar e se beneficiar da PrEP.^14^

Uma limitação importante deste estudo é que ele se baseia em dados secundários e anonimizados do Siclom, apresentada por meio do painel de PrEP,^8^ o que impede uma análise qualitativa mais aprofundada das razões que levam à baixa adesão e acesso à PrEP entre pessoas trans e travestis. Embora o estudo identifique disparidades relevantes, ele não fornece informações detalhadas sobre as barreiras sociais, culturais e estruturais enfrentadas por essa população. Além disso, a falta de dados censitários sobre a população trans no Brasil dificulta a estimativa precisa da cobertura da PrEP, o que é essencial para avaliar a efetividade das políticas públicas e do alcance desse método preventivo.^21^

Pesquisas futuras podem se beneficiar de metodologias qualitativas e mistas para compreender com mais profundidade os fatores que afetam o acesso e a permanência das pessoas trans nos programas de PrEP. Investigações que envolvam entrevistas com profissionais de saúde e usuários podem revelar aspectos relacionados à discriminação, acolhimento inadequado e falta de informação, que não são captados por dados quantitativos^19^. Além disso, estudos que analisem o impacto de intervenções específicas, como a prescrição de PrEP por farmacêuticos e enfermeiros, poderão contribuir para a ampliação das estratégias de implementação. Outra linha promissora seria explorar a introdução de novas formas de PrEP de longa duração, visando aumentar a adesão entre populações vulneráveis.^22^

Embora este estudo tenha apresentado contribuições ao revelar a baixa representatividade das pessoas trans entre os usuários de PrEP no Brasil, ele também reforça a necessidade de políticas mais inclusivas e direcionadas. A expansão do acesso à PrEP é fundamental para promover a equidade e reduzir a incidência de HIV nessa população, especialmente considerando as altas taxas de prevalência do vírus entre pessoas trans. Portanto, ampliar as estratégias de prevenção e enfrentar as barreiras estruturais e sociais são passos essenciais para garantir que a PrEP beneficie de forma mais efetiva aqueles que dela mais necessitam.
